# Apical size and *deltaA* expression predict adult neural stem cell decisions along lineage progression

**DOI:** 10.1126/sciadv.adg7519

**Published:** 2023-09-01

**Authors:** Laure Mancini, Boris Guirao, Sara Ortica, Miriam Labusch, Felix Cheysson, Valentin Bonnet, Minh Son Phan, Sébastien Herbert, Pierre Mahou, Emilie Menant, Sébastien Bedu, Jean-Yves Tinevez, Charles Baroud, Emmanuel Beaurepaire, Yohanns Bellaiche, Laure Bally-Cuif, Nicolas Dray

**Affiliations:** ^1^ Institut Pasteur, Université Paris Cité, CNRS UMR3738, Zebrafish Neurogenetics Unit, Team supported by the Ligue Nationale Contre le Cancer, Paris 75015, France.; ^2^ Sorbonne Université, Collège Doctoral, Paris F-75005, France.; ^3^ Institut Curie, Université PSL, Sorbonne Université, CNRS UMR 3215, Inserm U934, Genetics and Developmental Biology, Paris 75005, France.; ^4^ LPSM, Sorbonne Université, UMR CNRS 8001, Paris 75005, France.; ^5^ Institut Pasteur, Université Paris Cité, Physical Microfluidics and Bioengineering, Paris F-75015, France.; ^6^ LadHyX, CNRS, Ecole Polytechnique, IP Paris, Palaiseau 91120, France.; ^7^ Institut Pasteur, Université Paris Cité, Image Analysis Hub, Paris, France.; ^8^ Laboratory for Optics and Biosciences, CNRS, INSERM, Ecole Polytechnique, IP Paris, Palaiseau, France.

## Abstract

The maintenance of neural stem cells (NSCs) in the adult brain depends on their activation frequency and division mode. Using long-term intravital imaging of NSCs in the zebrafish adult telencephalon, we reveal that apical surface area and expression of the Notch ligand DeltaA predict these NSC decisions. *deltaA*-negative NSCs constitute a bona fide self-renewing NSC pool and systematically engage in asymmetric divisions generating a self-renewing *deltaA^neg^* daughter, which regains the size and behavior of its mother, and a neurogenic *deltaA^pos^* daughter, eventually engaged in neuronal production following further quiescence-division phases. Pharmacological and genetic manipulations of Notch, DeltaA, and apical size further show that the prediction of activation frequency by apical size and the asymmetric divisions of *deltaA^neg^* NSCs are functionally independent of Notch. These results provide dynamic qualitative and quantitative readouts of NSC lineage progression in vivo and support a hierarchical organization of NSCs in differently fated subpopulations.

## Teaser

The NSC lineage is reconstructed in association with a temporal series of predictive cellular and molecular hallmarks in situ.&nbsp;

## INTRODUCTION

Neural stem cells (NSCs) produce neurons and glial cells important for the physiology and plasticity of the adult vertebrate brain (*1*–*3*). To ensure these functions, NSC populations remain active and neurogenic during adult life. The efficiency of NSC population maintenance varies greatly between species and with age, and the mechanisms involved remain incompletely understood.

NSC population maintenance is the net result of two major fate decisions: NSC activation from quiescence (over time leading to NSC exhaustion) and the occurrence of self-renewing divisions. These decisions are actively studied in the telencephalic niches of the mouse and zebrafish adult brain [subependymal zone (SEZ) of the lateral ventricle and subgranular zone (SGZ) of the hippocampus in mouse and pallium in zebrafish], today the most tractable and comparable models to address NSC behavior in vivo (*4*, *5*). In both species, NSCs reside most of their time in the G_0_ quiescence state, under control of quiescence-promoting pathways (notably Notch2/3 and Bone Morphogenetic Protein (BMP) signaling) that gate G_1_ entry (so-called “activation”) in vivo (*6*). These factors are superimposed on cell-intrinsic windows of responsiveness to activation signals (*7*), which remain to be identified. Concerning the division mode, clonal tracing and intravital imaging in mouse and zebrafish highlight that NSCs can divide in a symmetric self-renewing (NSC/NSC), asymmetric (NSC/NP), or symmetric neurogenic (NP/NP) fashion where NP [“TAP” (transit amplifying progenitor) in mouse] is a nonglial intermediate progenitor committed to neuron generation after a few divisions. The balance between these distinct outcomes affects NSC maintenance amplification, steady state, and loss (*8*–*15*). These choices could be cell autonomous or involve some degree of cell-cell interactions, which could explain the coordination observed at the level of the population (*14*, *16*, *17*). In vitro, the NSC/NP division mode of adult mouse NSC involves the asymmetric expression of the Dyrk1a kinase, a regulator of Notch signaling (*18*, *19*). When the Notch ligand Delta-like1 is overexpressed, asymmetric NSC/NP division also correlates with the segregation of Delta-like1 in the NP daughter (*20*). In vivo, NSC populations at any time form a patchwork of asynchronous NSCs with distinct molecular states (*21*–*24*), morphologies (*25*, *26*), and fate (*10*, *14*, *16*, *27*), and regulators of NSC fate decisions within the intact neurogenic niche remain to be identified.

To identify mechanisms controlling NSC activation and fate decisions in vivo, we used intravital imaging to reconstruct adult NSC lineages and decipher cell-intrinsic features that characterize NSC decisions. Adult NSCs in the zebrafish adult pallium are radial glial cells (*25*), intermingled with NPs, covering the pallial surface. Because of this superficial position, one can record the behavior of all progenitor cells (NSCs and NPs) in their intact neurogenic niche, with single-cell resolution and over weeks (*8*, *14*, *15*, *28*). Thus, lineages can be captured at long term, allowing to monitor their progression despite the very slow time dynamics (*8*, *14*). To characterize pallial NSC lineage features, we performed long-term (up to 52 days) intravital imaging of the NSC population in a double transgenic context expressing a transcriptional reporter of *deltaA* (*29*), the most expressed Notch ligand in the adult pallium (*30*), and a novel reporter line for tight junctions to highlight apical NSC shapes. We optimized previous methods and analyzed about 1000 NSC tracks in situ, under physiological conditions or upon manipulation of apical surface area (AA) and Notch signaling, eventually allowing to link AA and *deltaA* expression with self-renewal, neurogenic potential, and lineage progression.

## RESULTS

### Cellular hallmarks quantitatively correlating with NSC states

**Fig. 1.**
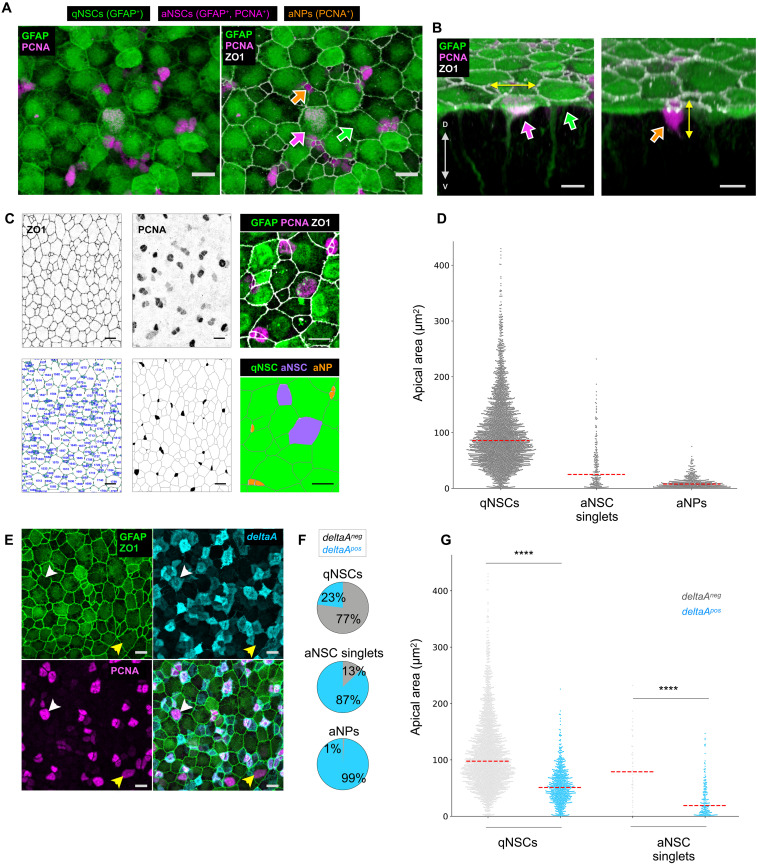
Apical area correlates with cellular types and states in the germinal zone of the adult zebrafish pallium. (**A** and **B**) High magnification of a 3-mpf *Tg*(*gfap:eGFP*) whole-mount pallium processed for IHC for GFP (green), PCNA (magenta), and ZO1 (white). Arrowheads to qNSCs (GFP^+^, green), aNSCs (GFP^+^ PCNA^+^, magenta), and aNPs (PCNA^+^, orange). (A) Dorsal view. ZO1 reveals the apical area (AA) of each cell and the cellular topology of the tissue. (B) Optical section perpendicular to the pallial ventricular zone, passing, from left to right, through a qNSC, an aNSCs, and a cluster of two aNPs. Double-headed white arrow to the direction of the dorsoventral axis, double headed yellow arrows to the main axis of elongation of the nucleus in an aNSC and an aNP. Scale bars, 10 μm. (**C**) Top: Photographs of whole-mount pallia (dorsal views, Dm); bottom: corresponding segmented views (green circle: cell vertex). Left: Segmentation of AA based on ZO1 (blue number: cell ID used for subsequent analysis). Middle: Segmented regions with detected marker, here PCNA (manually corrected segmentation to avoid false-positive cells). Right: High magnification of a preparation stained for the markers of interest with the corresponding final segmentation and cell identities (color coded). (**D**) Distribution of AAs of qNSCs, aNSCs, and aNPs in Dm for four brains pooled. Red dashed lines: median. (**E**) Maximum projection of a dorsal view of a whole-mount IHC in a 3-mpf *Tg*(*deltaA:eGFP*)*;Tg*(*gfap:dtTOMATO*) fish labeled for dTomato (green), ZO1 (green), eGFP (cyan), and PCNA (magenta). Arrowheads to a *deltaA^pos^* aNSC (white) and *deltaA^neg^* aNSC (yellow). Scale bars, 10 μm. (**F**) Proportion of *deltaA^pos^* cells (cyan) within the indicated cell types (*n* = 4 independent hemispheres, Dm). (**G**) Distribution of AAs in qNSCs and aNSC singlets according to *deltaA* expression. Red dashed line: median (*n* = 4 independent hemispheres, Dm). Statistics: Nonparametric *t* test (Mann-Whitney test), *P* < 0.0001.

Cell geometry regulates essential processes such as growth, lineage commitment, and signaling in various stem cell systems (*31*, *32*). In addition, apical cell shape in embryonic epithelia correlates with or determines cell fate (*33*–*35*). To characterize the organization of NSCs within their niche, we first assessed which cell state/type has an apical contact. We focused on the dorsal (Dm) and anterior (Da) pallial areas, which have been most extensively analyzed and are actively engaged in neurogenesis (*8*). We performed triple whole-mount immunohistochemistry (IHC) on adult pallia [3 months postfertilization (mpf)] from the *Tg*(*gfap:eGFP*) transgenic line (*36*) to label the tight junction protein zona occludens 1 (ZO1) together with NSCs [green fluorescent protein–positive (GFP^+^)] and cell proliferation [proliferating cell nuclear antigen (PCNA)] (Fig. 1A). This combination identifies quiescent NSCs (qNSCs) (GFP^+^, PCNA^−^), activated NSCs (aNSCs) (GFP^+^, PCNA^+^), and activated NPs (aNPs) (GFP^−^, PCNA^+^), while ZO1 delimits the apicobasal interface of polarized cells. Notably, PCNA is visible throughout the G_1_-S-G_2_-M phases of the cell cycle and shortly after the division event, resulting in doublets of aNSCs (*8*, *28*); thus, we only selected isolated aNSCs (aNSC singlets) to focus on aNSCs before division. We found that all three cell types display apicobasal polarity and contact the pallial ventricle through their apical surface. They are also in direct contact with each other through their ZO1-positive tight junction, forming a monolayer. Besides, we qualitatively observed that the nuclei of qNSCs and aNSCs are very close to and aligned with the pallial ventricular surface, whereas aNP nuclei are often found in a deeper position (Fig. 1B), in line with aNPs being on their way to delaminate upon differentiation into neurons. These observations highlight that the adult pallial germinal pool is organized as a pseudo-stratified neuroepithelial monolayer where the apical surfaces of qNSCs, aNSCs, and aNPs are spatially intermingled.

In contrast to most epithelia, where AAs are largely homogeneous, we observed that the AA of NSCs and their aNP progeny are highly heterogeneous in shape and size (Fig. 1A). This prompted us to probe for a possible implication of AA and associated parameters along lineage progression. We extracted quantitative information on various geometrical cell parameters of NSCs/NPs and determined whether they differ among cell types (NSCs versus NPs) or NSC states (qNSCs versus aNSCs). We segmented the cell contour of all progenitor cells on triple IHC of *Tg*(*gfap:eGFP*) fish at 3 mpf labeled with ZO1, PCNA, and *gfap*:GFP. We adapted a code previously developed for *Drosophila* epithelial tissues (*37*) and quantified AA, anisotropy, perimeter length, and the number of neighbors to correlate them with molecular markers (Fig.1C and fig. S1A). While the shapes of apical surfaces displayed a broad range of anisotropies across all cells of the germinal population, we found significant correlations of cell states and types with AA, apical perimeters, and the number of apical neighbors (Fig.1D and fig. S1A): On average, qNSC AAs are larger (84 μm^2^ ± 8.9 SD) than those of aNSC singlets (54 μm^2^ ± 17.1 SD) and aNP AAs are even smaller (10 μm^2^ ± 3.6 SD). The small AA of aNPs fits with their delaminating behavior (Fig. 1, B and C, orange, right column) and is the most discriminative morphological parameter compared to aNSCs (fig. S1, A and B).

Two nonexclusive hypotheses could underlie the observed correlation between AA and NSC state: (i) NSC geometry could influence NSC activation, or (ii) NSCs may have distinct proliferation rates modulating their geometry (e.g., their AA). The latter hypothesis is supported below.

### *deltaA* expression correlates with cell type, state, and quantitative apical parameters

Notch signaling promotes quiescence and progenitor state maintenance in adult NSCs in zebrafish and mouse (*7*, *30*, *38*–*40*). Thus, we also focused on this pathway as a further possible readout of NSC decisions in vivo. In the zebrafish adult pallium, *deltaA* expression is restricted to some NSCs and NPs (*8*, *30*), like in the adult mouse SEZ where aNSCs and TAPs express its ortholog *Delta-like 1* (*Dll1*) (*20*, *21*).

To achieve a quantitative description, we performed quadruple IHCs on double transgenic *Tg*(*gfap:dTomato*)*;Tg*(*deltaA:egfp*) fish to detect Tomato (*gfap*) (*41*), GFP (*deltaA*) (*29*), PCNA, and ZO1. We considered *deltaA^pos^* all cells with weak to strong IHC GFP signal, and *deltaA^neg^* all cells with no visible IHC GFP signal. We found that, in these static preparations, most qNSCs (77%) are *deltaA^neg^*, while most aNSC singlets (87%) and aNPs (99%) are *deltaA^pos^* (Fig. 1, E and F) (*8*). Next, we found a strong anticorrelation between AA and *deltaA* expression in NSCs: *deltaA^pos^* NSCs display on average a significantly smaller AA than do *deltaA^neg^* NSCs (*P* < 0.0001), both for all NSCs and for the qNSC and aNSC states separately (Fig. 1G and fig. S1C). *deltaA^pos^* qNSCs have on average smaller AAs (54 μm^2^ ± 3.7 SD) than their *deltaA^neg^* counterparts (112 μm^2^ ± 15.4 SD), and *deltaA^pos^* aNSCs (32 μm^2^ ± 8.3 SD) have smaller AAs than *deltaA^neg^* aNSCs (91 μm^2^ ± 28.2 SD). These observations reveal global trends between *deltaA* expression, a small AA, activation status, and lineage progression from NSC to NP. *deltaA* and AA may participate in or may read out lineage progression. They may be, or not, functionally interdependent factors in this process.

To help better understand causal links or the respective roles of AA and *deltaA* in NSC activation, we further explored NSCs that deviate from this general trend. First, there is a broad distribution of apical parameters at the level of individual NSCs. For example, 20% of qNSCs have an AA within the 5- to 60-μm^2^ range, corresponding to the average AA of aNSCs. Among aNSCs, 31% have an AA within the 50- to 150-μm^2^ range, corresponding to the average AA of qNSCs (Fig. 1D and fig. S1D). Second, a measurable fraction of cells also breaks the rule linking the *deltaA^pos^* and aNSC states: 23% of qNSCs are *deltaA^pos^* and 13% of aNSCs are *deltaA^neg^*. *deltaA^pos^* qNSCs have a small AA (around 50 μm^2^), while *deltaA^neg^* aNSCs have a large AA (above 50 μm^2^) (Fig. 1G and fig. S1C). These outliers reveal that the link between *deltaA* and AA (*deltaA^neg^*/small AA, *deltaA^pos^*/large AA) is stronger than the correlation between each parameter and NSC states.

### A novel transgenic tool and image analysis pipeline reveal NSC behavior in link with cell geometry and *deltaA* expression

**Fig. 2.**
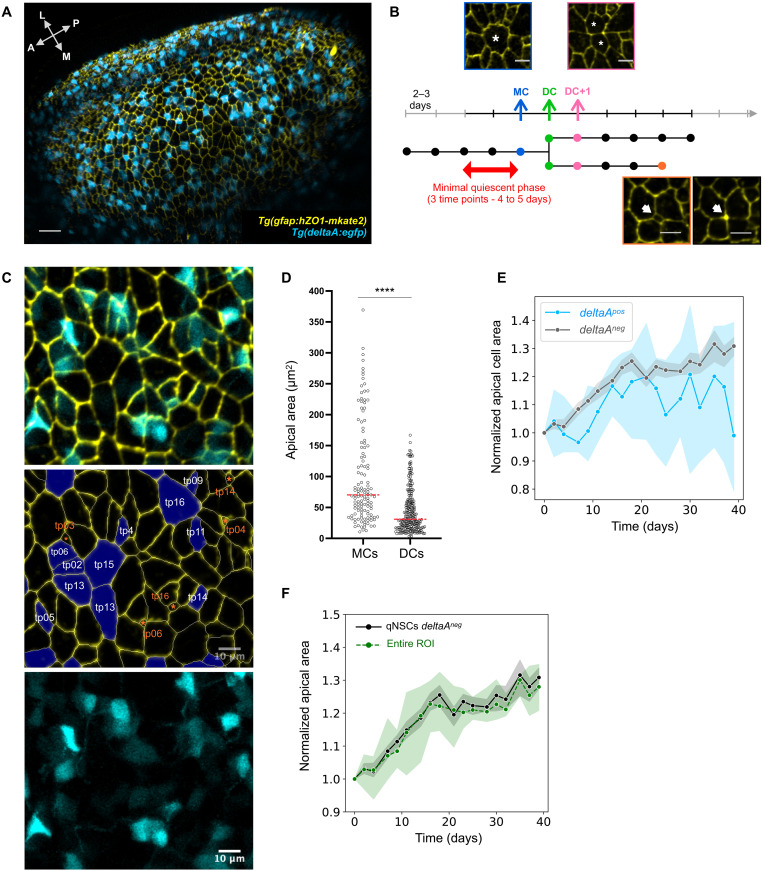
Apical area dynamics in NSCs. (**A**) 3D dorsal view of the pallial surface in a 2.5-mpf casper*;Tg*(*deltaA:eGFP*)*;Tg*(*gfap:hZO1-mKate2*) fish imaged intravitally using biphoton multicolor microscopy. *deltaA*:eGFP: *deltaA* transcription, cyan; *gfap*:ZO1-mKate2: NSC apical contours, yellow. Arrows show the anteroposterior (A-P) and mediolateral (M-L) axes. Scale bar, 30 μm. (**B**) Schematic example of a dividing track (horizontal tree) (horizontal arrow: time; vertical bars: imaging tp, consecutive tps separated by 2 to 3 days), to position a mother NSC (MC, blue arrow, example picture), daughter NSCs (DCs, cytokinesis event, green arrow), and DC+1 at the next tp (pink arrow, example picture). DC is the first tp where two daughters can be identified. We only scored dividing tracks where MC was preceded by at least 4 to 5 days without division (black part of time arrow). Track interruption before the end of the recording period corresponds to a delamination (example in orange). Scale bars, 10 μm. (**C**) 3D maximum projection with split and merged channels of the first tp of a time lapse. Color code for the overlay of middle image: NSCs that will divide (purple, tp of division), cells that will delaminate (orange, tp of delamination). The apparent spreading of *deltaA*:GFP (magenta) into neighboring cells results from their body ballooning beyond their apical surface (movie S1). (**D**) AA distribution of all MCs and all their DCs. Red dashed line: median (*n* = 4 independent hemispheres, Dm), 102 μm^2^ for MCs and 45 μm^2^ for DCs (*P* < 0.0001, Mann-Whitney). (**E**) Normalized AA of qNSCs during nondivision phases relative to time and *deltaA* expression (*deltaA^neg^* NSCs: gray, *deltaA^pos^* NSCs: blue) (with median and 95% CI). (**F**) Normalized area of the entire region of interest (ROI) over time (green) versus normalized AA of *deltaA^neg^* qNSCs during nondivision phases [gray, as in (E)] (with median and 95% CI).

To interpret the heterogeneities observed among NSCs and with NPs regarding AA and *deltaA* expression, we interrogated these parameters along NSC trajectories in real time. To retrieve fate-related cellular events (activation, division mode, and delamination) together with a quantitative resolution of NSC AAs using intravital imaging, we built a new transgenic line expressing a truncated version of human ZO1 (*42*) fused to the mKate2 fluorescent reporter (hZO1-mKate2) under control of the *gfap* promoter (*36*). Using multicolor two-photon microscopy and double transgenic *Tg*(*gfap:hZO1-mKate2*)*;Tg*(*deltaA:egfp*) fish crossed into the double mutant *Casper* background (transparent due to mutations in pigmentation genes) (Fig. 2A) (*43*), we performed live intravital imaging of 2.5-mpf adult fish every 2 to 3 days for at least 17 time points (tp) (43 days). We also developed an image analysis pipeline to extract dynamic quantitative measurements from the time lapses (fig. S2, A and B). The *Tg*(*gfap:hZO1-mKate2*) line successfully labeled the NSC apicobasal interface with an excellent signal to noise ratio, allowing us to follow cytokinesis events—which all occur perpendicularly to the plane of the NSC layer—as well as delamination events (Fig. 2B and movie S1). This live analysis confirmed highly variable *deltaA:gfp* expression intensities. For simplicity, we binned the GFP signal in four intensity scores (from 0 = no expression to 3 = strong expression), validated by quantitative pixel values (fig. S2, C to E). We also compared the values obtained for AAs in live and fixed samples and found them highly similar (*R*^2^ = 0.92; fig. S2, F and G). As an additional calibration, to focus on divisions originating from a previously quiescent NSC, we estimated the time needed from NSC activation onset (initiation of expression of the G_1_ phase markers PCNA or MCM5) to cytokinesis (identified monitoring ZO1-mKate2). We imaged two 3-mpf *Tg*(*mcm5:egfp*) (*28*);*Tg*(*gfap:hZO1-mKate2*) fish for over 39 days and estimated the activation-to-cytokinesis transition rate to be 0.326 days^−1^ [95% confidence interval (CI), 0.28 to 0.37], i.e., a median time transition duration of 2.1 days (fig. S3A). This fits our previous study based on *Tg*(*gfap:dTomato*);*Tg*(*mcm5:egfp*) animals (*8*). We estimated that, in at least 86% of division events, MCM5 is switched ON less than 6 days before division. Thus, only division events preceded by a minimum of two imaging time intervals (4 to 5 days) without division were considered to originate from a previously quiescent NSC.

Next, we fully analyzed time-lapse movies from four different *Tg*(*gfap:hZO1-mKate2*)*;Tg*(*deltaA:egfp*) fish and could follow the fate of 828 NSCs. Most of these NSCs (634 tracks, 76.6% of all) are resting in a long quiescent phase, i.e., not dividing nor delaminating during up to 43 days of imaging. One hundred ninety-four cells divided at least once, enabling to explore their cell lineage. Using the criteria above, 125 of these were activation and division events from quiescence (divisions following a qNSC to aNSC transition). Last, 97 delamination events were observed. Delaminating cells are the smallest cells tracked, with a median AA of 8 μm^2^ and 98% expressing *deltaA*, strongly suggesting that they are aNPs (fig. S3B). They usually express *deltaA* at highest levels, confirming our fixed data analyses on the link between AA and *deltaA* expression (fig. S3C). All tracks showing a division, a delamination, and/or a change in *deltaA* expression over time are shown in fig. S3D.

Hereafter, considering only divisions following a quiescence event, we will refer to the first tp after division as DC (daughter NSC) and respectively name the preceding and following tp MC (mother NSC) and DC+1 (one tp after the appearance of two DCs) (Fig. 2B). Successive imaging tp are then referred to as DC+2, DC+3, etc. These dataset and measurements put us in a position to explore the temporal and quantitative relationship of NSC quiescence exit, division, and fate with *deltaA* expression and AA dynamics.

### Cytokinesis events and slow growth during quiescence control NSC apical area dynamics

We first characterized the dynamics of NSC AAs over time to identify the events leading to AA changes. Generally, tracking individual NSCs from one tp to the next during nondivision phases did not reveal significant AA modifications (fig. S3D). Unsurprisingly, the major NSC AA remodeling events are divisions, each generating two DCs of equal AA, the sum of which approximates the initial size of the MC (Fig. 2D). Thus, each division leads to a decrease of AAs by half. This is expected to generate NSCs of smaller and smaller AAs through divisions, raising the question of how some NSCs reach a large AA. We therefore measured global apical expansion rates (tracking AA of nondividing NSCs during 40 days). For nondividing *deltaA^pos^* NSCs (*n* = 49), this rate is almost null and shows a high variability (Fig. 2E). In contrast, the AAs of *deltaA^neg^* NSCs (*n* = 584) are overall growing by 30% in 40 days (Fig. 2E). This affects *deltaA^neg^* NSCs of small and large AA. Although growth does not appear linear (Fig. 2E), it suggests that some daughters could eventually regain the size of their mother in more than 100 days, a duration compatible with our previous estimations of average quiescence durations in adult pallial NSCs (*14*). To further validate this observation, we reasoned that the AA growth of *deltaA^neg^* NSCs should be paralleled with a global growth of the segmented and tracked area, given that *deltaA^neg^* NSCs account for most NSCs overall (80%). We found that this was indeed the case (Fig. 2F). Together, these results indicate that NSC AAs vary as a consequence of abrupt decreases at division and, in the case of *deltaA^neg^* NSCs, compensatory slow growth during quiescence.

### The behavior of *deltaA^pos^* NSCs is biased toward proliferation and differentiation

**Fig. 3.**
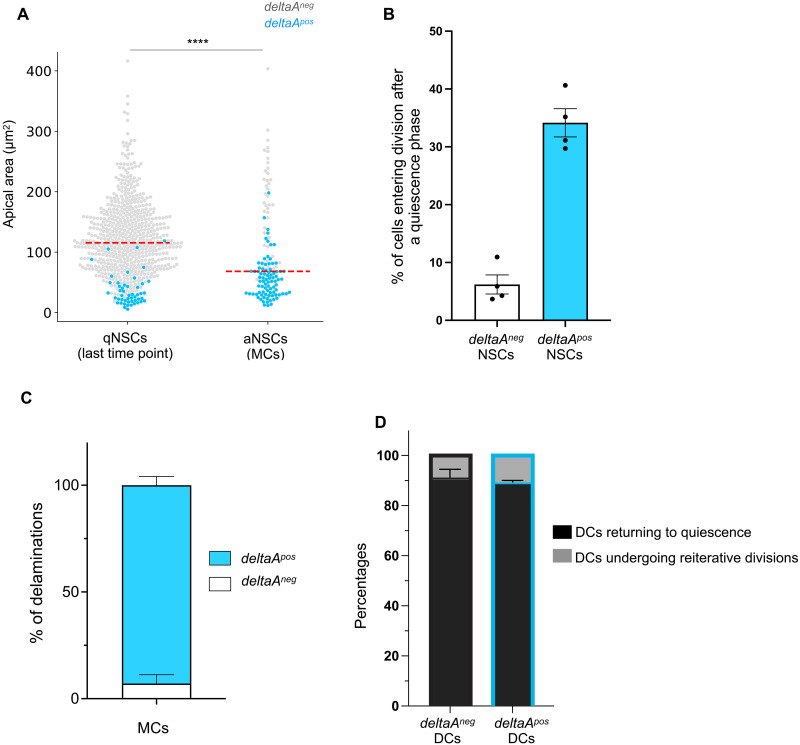
The behavior of *deltaA^pos^* NSCs is biased toward proliferation and differentiation. (**A**) Distribution of AA in qNSCs (measurements on the last tp of each time lapse) and aNSCs (measurements of MCs) according to *deltaA* expression (*n* = 4 independent fish pooled). Red dashed line: median. Statistics: Nonparametric *t* test between both distributions (Mann-Whitney test, two-tailed), *P* < 0.0001. (**B**) Percentages of *deltaA^pos^* versus *deltaA^neg^* NSC tracks where a post-quiescence division event takes place during a whole movie (*n* = 4 fish, median with 95% CI). (**C**) Percentages of delamination events occurring in *deltaA^pos^* MCs versus *deltaA^neg^* MCs (*n* = 4 fish, error bars for SEM). (**D**) Percentages of *deltaA^pos^* versus *deltaA^neg^* DCs returning to quiescence versus undergoing reiterative divisions after a post-quiescence division (*n* = 5 reiterative division events for *deltaA^neg^* DCs, *n* = 22 events for *deltaA^pos^* DCs, error bars for SEM).

We next assessed the overall dynamics of *deltaA* expression across time and NSC decisions. As in our static dataset (Fig. 1G), we confirmed a negative correlation between *deltaA* expression and AA, and we retrieved qualitatively and quantitatively similar qNSC and aNSC subtypes (Fig. 3A). In nondividing tracks (*n* = 703), *deltaA* expression appears continuous, with only 2% of NSCs changing their expression profile during a time lapse (nine NSCs switching *deltaA* OFF and five NSCs switching it ON). In dividing tracks (*n* = 125), *deltaA* expression before division is also largely stable, either always ON (77 tracks) or always OFF (40 tracks), with only 9.4% of dividing NSCs changing *deltaA* level before dividing (8 tracks switch *deltaA* ON before division out of 85 dividing *deltaA^neg^* MCs, and a single *deltaA^pos^* track switches *deltaA* OFF) (fig. S3D). Last, after division, when present in (a) daughter cell(s), *deltaA* expression persists in most cases for the remainder of the track (96.2% ± 1.9 SEM) (fig. S3D). Thus, *deltaA* expression is not a transient state but signs a stable change of NSC signature.

We then compared the proliferative and fate behavior of *deltaA^pos^* and *deltaA^neg^* NSCs (considering as *deltaA^pos^* all NSCs where GFP is visible). First, we found that among 168 *deltaA^pos^* NSCs, on average, 34.2% (±2.4% SEM) activate from quiescence and divide during a movie, which is around 5.5 times more frequently than *deltaA^neg^* NSCs (6.2% ±1.6% SEM, *n* = 628) (Fig. 3B). These values are confirmed using growth rates: *deltaA^neg^* NSCs, in average, have a growth rate of 5.587 × 10^−3^ day^−1^ (doubling time = 124 days), against 2.460 × 10^−2^ day^−1^ (doubling time = 28 days) for *deltaA^pos^* NSCs. Second, we found that the vast majority of delaminations in dividing tracks occur in the progeny of *deltaA^pos^* NSCs (92.8% ± 4.1 SD, *n* = 45 delaminations) (Fig. 3C). Third, our data also capture that the large majority of *deltaA^pos^* DCs (223 over 250 DCs tracked for at least 4 days after division) reenter quiescence after division, a proportion similar to that of *deltaA^neg^* DCs (Fig. 3D)—quiescence duration is, however, shorter in the case of *deltaA^pos^* DCs (see below).

Thus, although *deltaA* expression is neither necessary for division nor a criterion for immediate division, the fate of *deltaA^pos^* NSCs is biased toward proliferation and lineage termination. This bias is visible at long term, as delamination and differentiation can occur days to weeks after *deltaA* expression onset and involve several further NSC divisions and quiescence phases. The association of *deltaA* expression to an NSC state that is engaged at long-term toward neurogenesis correlatively associates the *deltaA^neg^* state with a signature of NSC stemness.

### The onset of *deltaA* expression signals the first asymmetric NSC division along the NSC lineage

**Fig. 4.**
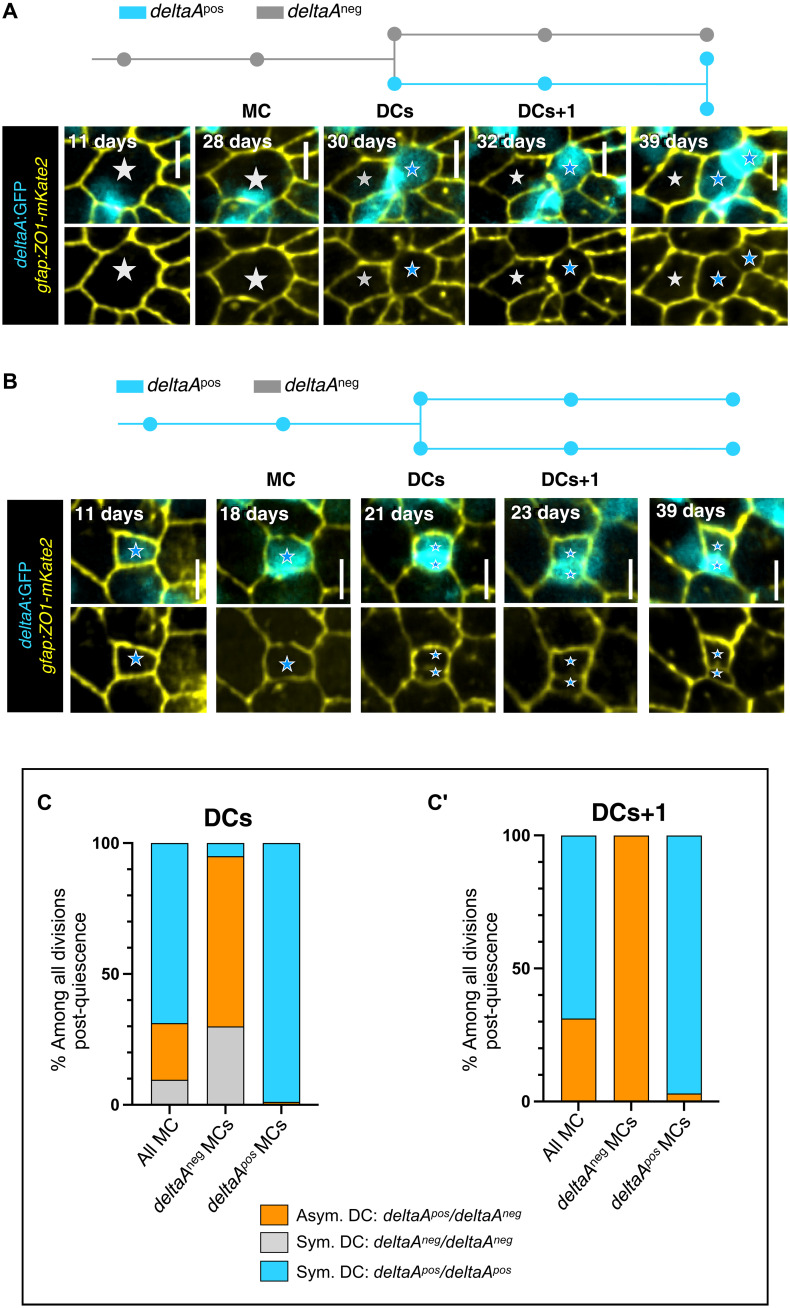
The *deltaA* expression status of NSCs predicts different division modes. (**A** and **B**) Examples of asymmetric (A) and symmetric (B) divisions based on *deltaA* expression. In each case, the track (*deltaA^pos^* NSCs: blue; *deltaA^neg^* NSCs: gray) corresponds to the representative snapshots displayed underneath, showing *gfap*:ZO1-mKate2 (yellow) and *deltaA*:GFP (cyan) (top) or *gfap*:ZO1-mKate2 only (bottom). Stars to MC and its DCs (gray stars: *deltaA* OFF, blue stars: *deltaA* ON). (**C** and **C′**) Percentages of each division mode observed for DCs (C) and DCs+1 (C′) (asymmetric *deltaA^neg^*/*deltaA^pos^*: orange; symmetric *deltaA^neg^*/*deltaA^neg^*: gray; symmetric *deltaA^pos^*/*deltaA^pos^*: blue) depending on the *deltaA^pos^* or *deltaA^neg^* status of the MC.

The investigation of *deltaA* expression dynamics first revealed that *deltaA* expression onset tightly correlates with cytokinesis. In all 40 division events of *deltaA^neg^* NSCs, *deltaA* transcription is initiated after division, in DCs or DCs+1 (fig. S3D). A second major observation was that dividing *deltaA^neg^* NSCs systematically generate daughter cells of opposite *deltaA* expression status, one NSC daughter remaining *deltaA^neg^*, while the other becomes *deltaA^pos^* (Fig. 4, A and C). This asymmetric outcome (ON/OFF, referred to below as “binary asymmetry”) can be already apparent for DCs and/or reinforced for DCs+1 as *deltaA* expression progressively becomes detectable in DC pairs that were initially *deltaA^neg^/deltaA^neg^* at DC (Fig. 4C′).

In marked contrast, *deltaA^pos^* NSCs systematically divided to initially generate two *deltaA^pos^* daughter NSCs (Fig. 4, B and C). Ranking *deltaA* expression using intensity scores (fig. S2, C to E) further revealed that DCs in ON/ON pairs often differ in GFP intensity (fig. S4, A and B). A fraction of these intensity-based asymmetries transformed into ON/OFF binary asymmetries over time, in average after 11 to 12 days (fig. S4C). Last, we determined whether the anti-correlation between AA and *deltaA* expression was detectable at cytokinesis when DCs have different sizes. We found that 60% of DC pairs have AAs that differ by at least 20% and that this increases to 76 and 82% of DCs+1 and DCs+2 pairs, respectively (fig. S4D, red curve). Among such DC pairs, a higher *deltaA* expression level in the smallest DC is the predominant situation, although this is acquired over time and 50% DC pairs initially display equal *deltaA* levels (fig. S4D).

Together, the onset of *deltaA* expression is largely slave to an NSC division, and most divisions of NSCs activating from quiescence generate daughters of different *deltaA* expression intensities over time. The divisions of *deltaA^neg^* MCs are the first asymmetric divisions of the lineage, generating a *deltaA^neg^*/*deltaA^pos^* pair of differentially fated DCs (stemness versus neurogenesis commitment, respectively). This asymmetry is established at or immediately after division; thus, it might depend on cell-cell interactions or contextual cues in DCs.

### AA and *deltaA* transcription before division are robust and independent predictors of activation propensity and binary *deltaA* asymmetry in DC pairs

**Fig. 5.**
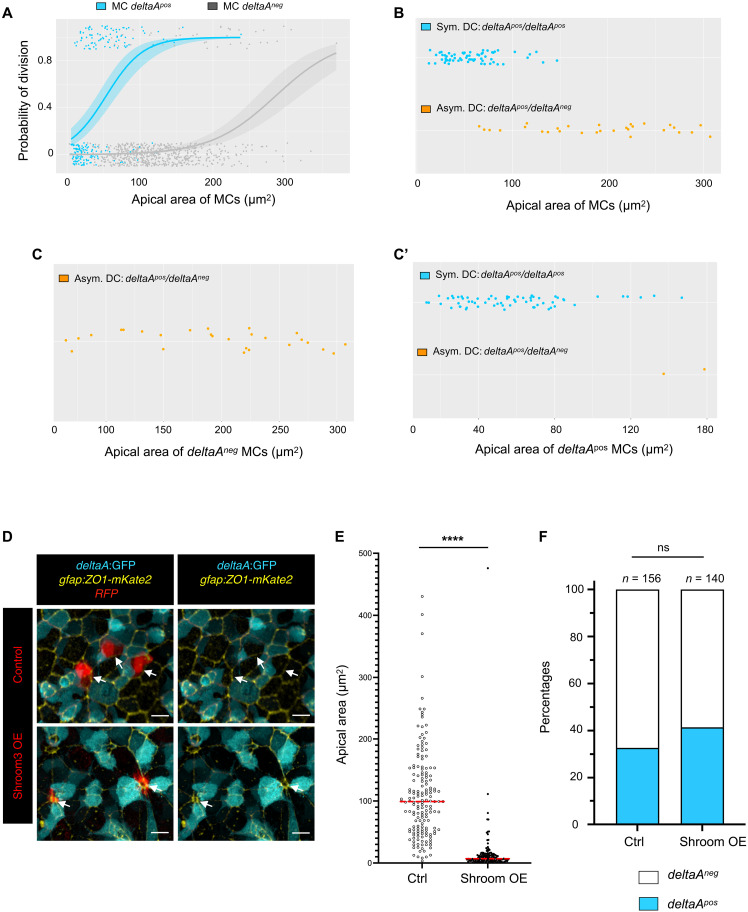
*deltaA* expression and AA individually predict NSC fate decisions. (**A**) Logistic regression model using AA and *deltaA* expression as covariates, showing the probability of NSC division as a function of AA for each *deltaA* expression status (*deltaA^neg^* MC: gray, *deltaA^pos^* MC: blue). The statistical interaction between AA and *deltaA* was found statistically significant (type II Wald χ^2^ tests, *P* = 4.67 × 10^−3^). (**B** to **C′**) *deltaA* expression status in DC+1 pairs (symmetric *deltaA^pos^/deltaA^pos^* or asymmetric *deltaA^neg^/deltaA^pos^*) as a function of the apical area of MCs, (B) when all MCs are considered together (*deltaA^pos^/deltaA^pos^* pair: blue, *deltaA^neg^/deltaA^pos^* pair: orange), or (C) when *deltaA^pos^* (blue) and (C′) when *deltaA^neg^* (gray) MCs are considered separately. (**D**) High magnification of the NSC layer showing *shroom3*-electroporated NSCs (Shroom3-mCherry, bottom row; control expressing mCherry only, top row) in 3-mpf *Tg*(*deltaA:egfp*) fish immunostained for GFP (cyan) and ZO1 (yellow). (**E**) Effect of Shroom3 overexpression on NSC AA. Shroom3-mCherry–overexpressing NSCs: *n* = 148, mean AA: 15 μm^2^; control electroporated NSCs: *n* = 167, mean AA: 109 μm^2^. Statistics: Nonparametric *t* test (Mann-Whitney test), *P* < 0.0001. (**F**) Effect of Shroom3 overexpression on *deltaA* expression. Proportion of *deltaA^pos^* sNSCs among electroporated qNSCs: Shroom3-dsRed overexpression: 32% (*n* = 140); dsRed overexpression (control): 41% (*n* = 156). Statistics: Two-sided Fisher’s exact test, *P* value ns (not significant).

AA and *deltaA* expression are strongly correlated with each other, and with NSC division frequency and division mode over time. Thus, we wondered whether these two parameters are predictors of NSC decisions and, if so, whether one parameter dominates. To first evaluate the predictive value of AA and/or *deltaA* expression before division on activation frequency, we conducted logistic regressions to evaluate the probability of division for qNSCs as a function of NSC AA for all tracks. In both *deltaA^neg^* and *deltaA^pos^* qNSCs, activation and division probability increased with AA (Fig. 5A). For a given AA, this probability was higher in *deltaA^pos^* NSCs (Fig. 5A). Considering *deltaA* expression levels further shows that, for a given AA, high expression scores are associated with a higher division propensity than low scores (fig. S5A). However, size alone, in the absence of the *deltaA* expression parameter, is not sufficient to predict the probability of NSC division (fig. S5B).

We next addressed the predictability power of AA and *deltaA* expression on NSC division asymmetry, focusing on the generation of *deltaA^neg^*/*deltaA^pos^* DC+1 pairs. This binary asymmetrical outcome appears to be highly predicted by AA when all cells are considered (Fig. 5B). However, size becomes an irrelevant parameter when *deltaA^neg^* MCs are considered separately: Irrespective of their AA, *deltaA^neg^* mothers divide in a binary asymmetric manner (100% of DCs+4 cases, *n* = 27) and *deltaA^pos^* mothers generate *deltaA^pos^*/*deltaA^pos^* DC pairs (97% of DCs+4 cases, *n* = 66) (Fig. 5, C and C′).

Together, these results indicate that the nonexpression of *deltaA* systematically predicts the generation of asymmetrically fated DCs upon division. It also predicts a lower division frequency overall than for NSCs expressing *deltaA*, while a large AA biases division propensity toward higher division rates for both the *deltaA^neg^* and *deltaA^pos^* NSC types.

### NSC AA and Notch signaling are functionally independent parameters in the control of NSC decisions along lineage progression

We next addressed whether and to which extent *deltaA* and AA may be functionally interacting parameters. To address the effect of AA on *deltaA* expression, we overexpressed the PDZ domain–containing protein Shroom3, or its dominant-negative form dnShroom3, using intracerebral injection and electroporation of Shroom3-mCherry– or dnShroom3-mCherry–encoding plasmids into pallial NSCs of *Tg*(*deltaA:egfp*) adults in vivo. These factors were reported to decrease versus increase AA in embryonic epithelial cells (*35*). While dnShroom3 was without effect on NSC AA in our system, Shroom3 led to extremely efficient AA shrinkage within 3 days after electroporation, as measured using ZO1 IHC (Fig. 5, D and E). This was not associated with a significant induction of *deltaA* expression in electroporated qNSCs (41% *deltaA^pos^* in Shroom3 overexpression versus 32% in control, *n* = 296 qNSCs counted in 17 brains) (Fig. 5F).

**Fig. 6.**
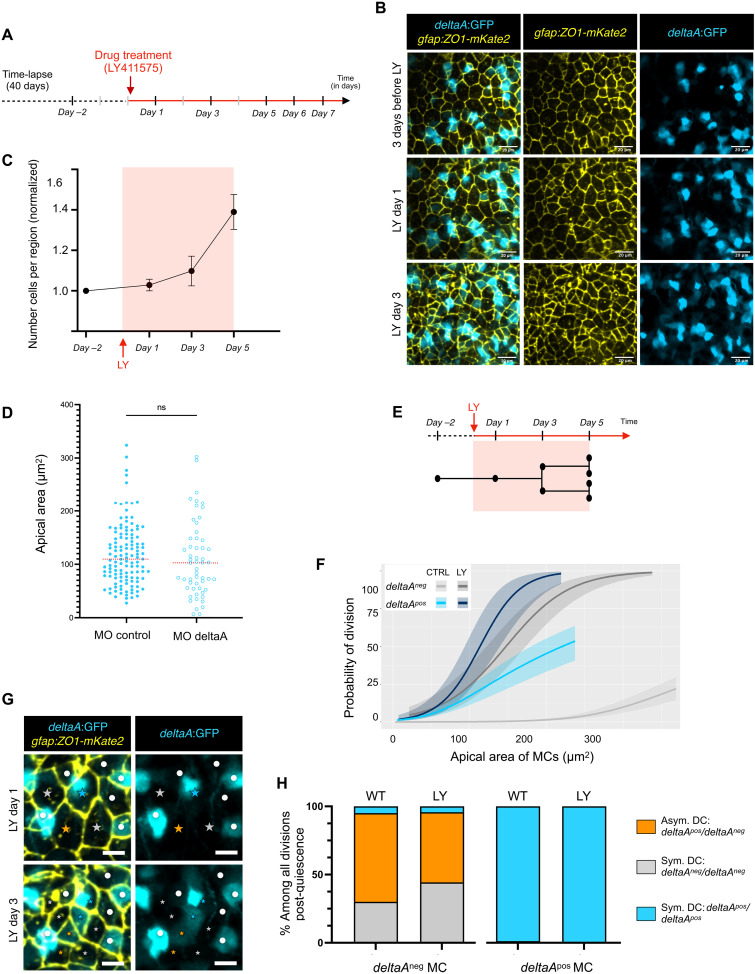
*deltaA* expression and AA in mother NSCs predict NSC decisions independently of Notch signaling. (**A**) LY was added to the fish water for 7 days. Fish were imaged at days 1, 3, 5, 6, and 7 of treatment. (**B**) Merged and split snapshots, maximum projections (dorsal views) of a live-imaged 3-mpf casper*;Tg*(*deltaA:eGFP*)*;Tg*(*gfap:hZO1-mKate2*) fish showing progressive NSC reactivation upon LY treatment (*gfap*:ZO1-mKate2: yellow, *deltaA*:GFP: cyan). Scale bars, 20 μm^2^. (**C**) Number of cells per segmented region (*n* = 3 fish, mean and error bars for SEM), normalized to the value at day −2, as a function of time [see (A)]. (**D**) Electroporation of *deltaA*-MO in Tg(*gfap*:dtTomato) shows no effect on the AA of GFAP^pos^ cells (control MO: *n* = 123 cells, *deltaA*-MO: *n* = 55 cells). Statistics: Nonparametric *t* test (Mann-Whitney), *P* value ns, median in red. (**E**) Time window of reliable NSCs tracking upon LY treatment. (**F**) Logistic regression: Probability of NSC division as a function of AA for each MC *deltaA* status; compared untreated (CTRL) and LY conditions. Notch inhibition abolishes the differences in activation rate between *deltaA^pos^* and *deltaA^neg^* NSCs (*P* = 0.16, χ^2^ test), but the AA effect persists. Predictions for CTRL: Rescaling of the rates over 4 days from all divisions over the duration of the movies; predictions for LY: estimated using all divisions over 4 days (see fig. S6A). Individual regressions pooled for this analysis: see (E). (**G**) Merged and split time-lapse images of a live-imaged 3-mpf casper*;Tg*(*deltaA:eGFP*)*;Tg*(*gfap:hZO1-mKate2*) fish highlighting dividing NSCs (colored stars to MCs and their progeny) and nondividing NSCs (white dots) during the first and third days of LY treatment. Scale bars, 10 μm^2^. (**H**) Division modes based on *deltaA* expression in DCs, for divisions occurring after 3 days of LY (*n* = 3 fish) or in CTRL (*n* = 4 fish).

Next, we indirectly manipulated DeltaA activity by perturbing Notch signaling using the γ-secretase inhibitor LY411575 (LY) (*7*, *8*). This treatment has the advantage of blocking signaling in both *deltaA^pos^* NSCs and their neighbors. To permit a dynamic analysis with knowledge of NSC history, two of the *Casper;Tg*(*gfap:hZO1-mKate2*)*;Tg*(*deltaA:egfp*) adult fish initially subjected to intravital imaging for 40 days and an additional one fish imaged at this last tp were treated with LY while imaging for a further 7 days. Recording was conducted at days 1, 3, 5, 6, and 7 after treatment onset (Fig. 6, A and B), and we reconstructed NSC trajectories (261 tracks under LY treatment) (see fig. S6A for all dividing tracks). Blocking Notch primarily activates NSCs (*7*, *8*, *30*). We observed a first massive wave of induced cytokinesis on the third and fifth day of treatment (Fig. 6, B and C), validating our Notch blockade procedure, here concomitant with imaging: 89 NSCs divided among 261 NSC tracked. This is 19 times more than the overall likelihood of NSCs to divide under control conditions (per cell per day, upon LY treatment: 0.0852, 95% CI, 0.0715 to 0.1001 versus control: 0.0044, 95% CI, 0.0038 to 0.0052). We found that Notch blockade was not accompanied with changes of AA during this time frame: The AA of nondividing NSCs was stable and dividing NSCs generated DCs of half the size of their mother’s AA (fig. S6, A and B). To directly challenge the role of DeltaA in these observations, we knocked down *deltaA* using a fluorescein-tagged morpholino (MO) electroporated into NSCs upon intraventricular injection into the adult brain in vivo. This MO was chosen to target the *deltaA* transcription start site (*44*), present in the *deltaA:egfp* transgene. Electroporation into adult *Tg*(*deltaA:egfp*) fish validated a significant decrease of GFP expression at 3 days after electroporation (fig. S6C). Similar electroporations into *Tg*(*gfap:dTomato*) fish also lead to a significant decrease in NSC activation (fig. S6D). We observed, however, that NSC AA was not modified upon *deltaA* knockdown at 3 days after electroporation (Fig. 6D), as observed with Notch signaling blockade.

We next used this paradigm to test whether the prediction power of AA on division propensity in vivo resists a context where Notch signaling is blocked. We focused on the first 5 days of treatment, as the numerous NSC division events made it difficult to faithfully connect DCs to their MC afterward (Fig. 6E). During the first 5 days of treatment, the initial expression of *deltaA:gfp* in NSCs was largely unaffected, allowing to unambiguously track both *deltaA^neg^* and *deltaA^pos^* NSC categories. Logistic regressions measuring NSC division frequency as a function of AA confirmed that the predictive character of AA on division propensity can be detected over a period of 4 days in control *deltaA^neg^* and *deltaA^pos^* NSCs (Fig. 6F). Upon LY treatment, AA still appeared predictive of division propensity in both cell categories, which now displayed identical regression profiles shifted toward higher activation rates (Fig. 6F and fig. S6E). Thus, under physiological conditions, both *deltaA^neg^* and *deltaA^pos^* NSCs are limited in their activation frequency by ongoing Notch signaling (although at different levels or with different sensitivities). Moreover, the predictive character of AA on division propensity operates in the absence of Notch signaling. This reveals the existence of a process biasing activation rate in a manner independent from the Notch signaling level or status of the NSC considered.

Last, given the identified predictive power of *deltaA* expression on division mode, we addressed whether Notch signaling was involved in controlling NSC fate at division. To enrich our analysis for NSC divisions that occurred as a result of LY, we focused on DC pairs revealed after 3 days of LY treatment, i.e., with their single MC detectable at 1 day of treatment (Fig. 6G). Control animals were analyzed over the same duration. We could record all three division modes, generating *deltaA^neg^*/*deltaA^neg^*, *deltaA^neg^*/*deltaA^pos^*, and *deltaA^pos^*/*deltaA^pos^* DC pairs (Fig. 6, G and H). We specifically focused on divisions from *deltaA^neg^* MCs. While their exclusive *deltaA^neg^*/*deltaA^pos^* division fate is very rapidly acquired after cytokinesis, around 30% of the DC pairs solidify this fate between DC and DC+1 from an initially *deltaA^neg^*/*deltaA^neg^* fate (Fig. 4, C and C′). We observed no difference in the DC fate of *deltaA^neg^* MCs under Notch blockade compared to control conditions (Fig. 6H) and, as previously concluded, AA appeared irrelevant (fig. S6F). Although fate consolidation could not be studied, these results suggest that the initial steps of asymmetry generation in the NSC lineage are independent of Notch signaling. We also observed no change in the *deltaA^pos^*/*deltaA^pos^* DC outcome of divisions from *deltaA^pos^* MCs (fig. S6F).

## DISCUSSION

Activation frequency and division modes condition NSC renewal and lineage progression, the two concomitant hallmarks of stemness. To understand how these parameters are controlled in vivo, we exploited long-term intravital imaging of NSC morphometric, molecular, and fate readouts to identify predictors of NSC decisions in the endogenous context of the intact neurogenic niche. We identify AA and *deltaA* expression as associated though functionally unlinked parameters that correlate with NSC division propensity. We further uncover the first detectable asymmetry in the NSC lineage, where *deltaA^neg^* NSCs systematically generate daughter NSCs that effectively segregate self-renewal and neurogenesis commitment. Our results provide the first NSC lineage reconstruction associated with a temporal series of predictive cellular and molecular hallmarks in situ. These hallmarks underscore the relevance of intrinsic cues associated with the *deltaA^neg^* status in the accomplishment of stemness, a conclusion reinforced by our demonstration that these cues are independent of Notch signaling.

### A model of NSC lineage progression based on AA and *deltaA* expression

**Fig. 7.**
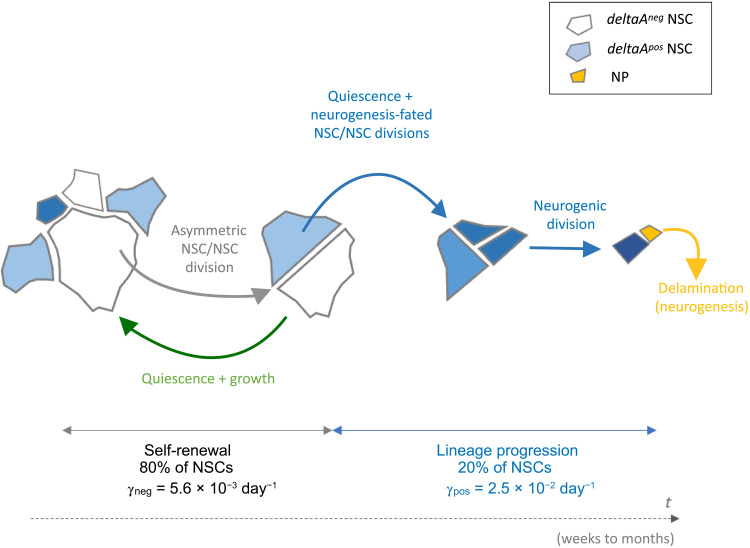
Dynamic hierarchy of self-renewal and lineage progression based on AA and *deltaA* expression in adult pallial NSCs in situ. Schematic apical representations of NSCs and NPs, with relative AAs and *deltaA* expression (color coded), in an interpretative drawing resulting from the assembly of overlapping tracks, covering a time frame of weeks to months. The division of *deltaA^neg^* NSCs signs the transition from self-renewal to neurogenesis commitment. γ_neg_ and γ_pos_ are average activation rates for *deltaA^neg^* and *deltaA^pos^* NSCs, respectively.

The transgenic line *Tg*(*gfap:ZO1-mKate2*) reveals the dynamics of NSC apical membranes, now allowing to investigate multiple individual cell or collective features such as apical cell geometries or neighborhoods, over different numbers of cells, in link with cell decisions (such as cytokinesis or delamination) and molecular markers (here, *deltaA*). Tracking for at least 40 days the behavior of >800 NSCs in their endogenous niche inside the adult zebrafish pallium (*8*, *28*), we could reconstruct a presumptive lineage spanning months of an NSC’s life. Focusing on cell-intrinsic features, we show that AA and *deltaA* expression, together, sign NSC position along lineage progression (Fig. 7): (i) the divisions of *deltaA^neg^* NSCs generate *deltaA^pos^* NSCs, placing *deltaA^neg^* NSCs hierarchically upstream; (ii) NSC AAs grow slowly over time together with an increasing probability to divide; (iii) conversely, high *deltaA* levels and a small AA sign temporal proximity to NSC pool exit and neuronal differentiation. In between these extremes, we identify a key lineage transition linked to both parameters and affecting fate: the division of *deltaA^neg^* NSCs. This event systematically generates differently fated daughter NSCs, of which the *deltaA^neg^* daughter has the potential to regrow its AA and behave identically to its mother, while the *deltaA^pos^* daughter does not regrow and engages into more frequent subsequent divisions and ultimately differentiation. Together, these results, supported by quantitative measures of division rates and AA growth, allow inferring relative temporal and hierarchical NSC states from in vivo images and interpreting static datasets staining NSCs in their niche.

### AA reads out activation propensity in adult NSCs

We previously identified Notch3 signaling as a major block of NSC activation and postulated a lateral inhibition–like mechanism originating at least from *deltaA^pos^* NPs (*8*). We now show that *deltaA^pos^* NSCs also receive a proliferation-inhibiting Notch signal, as attested by their increased propensity for activation upon Notch blockade (Fig. 6F). Last, the fact that NSCs receiving the *deltaA*-MO decrease their division propensity (fig. S6D) suggests that DeltaA may additionally act in cis to lower Notch signaling in their expressing NSCs.

In addition, our results point to AA as a novel cellular readout correlated with NSC division propensity. In embryonic radial glia, AA varies dynamically with nuclear position and cell cycle phase (*45*). The adult pallium is very different, however, as there is no interkinetic nuclear migration, and NSC AA during quiescence is either stable (in *deltaA^neg^* NSCs) or growing at a steady rate over weeks (in *deltaA^pos^* NSCs) (Fig. 2E). We also did not notice AA increase before division (fig. S3D). Our work does not attempt to solve the mechanisms leading to AA increase, although the observed growth rate suggests an active process rather than a passive phenomenon, e.g., due to stretching to accommodate size changes in neighbors.

In the embryonic retina, experimentally enlarging AA via dnShroom3 favors the proliferating over differentiating neural progenitor fate, in a process mediated by enhanced Notch signaling (*35*). Overexpression of dnShroom in adult NSCs in vivo did not enlarge AA, while, as observed in embryos, Shroom overexpression triggered massive AA reduction and delamination, precluding to directly probe AA impact on division frequency. Whichever mechanisms link AA and division propensity, our current results argue that this mechanism is Notch independent, because we did not observe an induction of *deltaA* expression in Shroom3-overexpressing NSCs (Fig. 5, D to F), AA was unchanged upon deltaA knockdown (Fig. 6D), and the link between AA and division propensity persists even upon LY treatment (Fig. 6F).

AA may be per se informative or read out another correlated geometry or subcellular organization feature (fig. S1). NSCs also have a large basolateral component, which branches over several hundreds of micrometers in the parenchyma and could receive or encode division-related signals (*46*, *47*). AA, however, appears as a hub that at least reads out pertinent information relative to NSC division propensity, within a given *deltaA* expression status. In *deltaA^neg^* NSCs, AA growth between divisions implies that, in a given cell, AA positively correlates with time in quiescence, which might be one of the measured parameters. There is, however, no threshold below or above which *deltaA^neg^* NSCs will systematically divide or remain quiescent, and further mechanistic work is needed to interpret this correlation. In the case of *deltaA^pos^* cells, 100% of cells with an AA of <10 μm^2^ delaminate during a 43-day movie (fig. S3F). A hierarchical relationship between AA decrease and fate acquisition remains to be studied, but Shroom3 overexpression indicates that delamination can also be induced by AA decrease in the absence of a fate change (as read by *deltaA*) (Fig. 5F).

Last, our dynamic intravital imaging results shed light on the mechanisms that account for the correlation between AA and *deltaA* expression in the adult pallial NSC population. At any time, static images show that *deltaA^neg^* NSCs are generally large and *deltaA^pos^* NSCs are generally small (Fig. 1). This is highly reminiscent of the checkerboard pattern modeled in embryonic neuroepithelia and interpreted to result from dictating Notch signaling directionality by the surface of contact between signaling and receiving cells (*48*). In the adult NSC population, where cell divisions and AA changes occur, our results support a distinct interpretation where AA differences between *deltaA^neg^* and *deltaA^pos^* NSCs emerge from two parameters: a regulation of *deltaA* transcription onset by lineage progression, coupled with the lower division frequency of *deltaA^neg^* NSCs and their AA regrowth during quiescence.

### The restriction of NSC potential is progressive, and stemness is signed by the *deltaA^neg^* status

Clonal tracing and intravital imaging revealed that adult NSCs can divide according to three possible division modes (NSC/NSC, NSC/NP, or NP/NP) in mouse and zebrafish (*8*–*15*). This classification is based on the generation of the NP terminal fate, and it is a major question to understand whether all NSCs are equal along lineage progression until this fate decision (*12*, *14*, *17*). Our results highlight asymmetric and overall generally increasing *deltaA*:GFP levels at each division of a *deltaA^pos^* mother (fig. S3C). Although these observations await confirmation by monitoring DeltaA protein—we currently lack an antibody detecting DeltaA in adult zebrafish NSCs, they suggest that sister *deltaA^pos^* cells are generally not equivalent and that fate acquisition is a progressive process along the division sequence of each *deltaA^pos^* NSC. This is highly reminiscent of the progressive transition from commitment to differentiation described in mouse skin stem cells (*49*). There was a trend but no clear-cut AA-related rule associated with the assignment of *deltaA*:GFP differences between daughters (fig. S4D). In addition, the massive induction of proliferation by LY treatment precluded analyzing *deltaA*:GFP levels between *deltaA^pos^* daughters in the absence of Notch. Last, assessing the role of DeltaA itself in lineage progression will necessitate intravital imaging upon long-term *deltaA* blockade, which was beyond the scope of this work. Together, the mechanisms involved in lineage progression and progressive fate restriction within the *deltaA^pos^* lineage remain open.

Our work also reveals that *deltaA^neg^* NSCs systematically generate daughters of opposite *deltaA* status. While the *deltaA^pos^* NSC engages toward a neurogenic fate at long term, several arguments suggest that the *deltaA^neg^* daughter behaves identically to its mother: It will never turn on *deltaA* expression (fig. S3D), it has the potential to regrow to the initial mother size (Fig. 2E), and all *deltaA^neg^* NSCs, whatever their size, follow this asymmetric division mode (Fig. 4, C and C′). These observations show that the *deltaA^neg^* daughter is engaged in self-renewal and identify this NSC/NSC division as the first asymmetric division of the NSC lineage, generating two NSC daughters of different potential that segregate stemness maintenance from neurogenesis. A possibly equivalent cell type, showing self-renewal at the individual cell level, was described in the adult mouse DG niche (*50*). The activation dynamics and downstream neurogenesis output of this cell type decrease with age (*51*), a point that remains to be formally addressed for *deltaA^neg^* NSCs in the zebrafish pallium. The systematic outcome of *deltaA^neg^* NSC divisions likely implies a cell-autonomous process and raises the question of its control. The *deltaA* status is tracked using the *Tg*(*deltaA:gfp*) transgene (*29*), implying transcriptional regulation of *deltaA* expression after division. Asymmetric segregation of the DeltaA protein itself could be directly monitored in embryonic neural progenitors in vivo (*44*, *52*) and in adult NSCs overexpressing Dll1-GFP in vitro (*20*). This is unlikely to drive the first NSC/NSC asymmetry identified here for the pallial lineage, because we do not detect *deltaA*:GFP expression before division. Ascertaining this point will nevertheless require the direct detection of DeltaA. Last, we found that the *deltaA* expression asymmetry is initially insensitive to Notch blockade (Fig. 6H). This argues against a mechanism such as intralineage regulation, involving Notch-mediated sister-sister interactions that can occur downstream of the asymmetric segregation of Notch pathway regulators other than Delta (*53*–*55*).

### Integration of NSC heterogeneity modalities

Heterogeneities in NSC potential are postulated on the basis of single-cell transcriptomics, 5-bromo-2′-deoxyuridine (BrdU) or genetic fate tracing, and intravital imaging (*5*). The temporal interpretation of the former approaches is inferred from statistical analyses at successive tp, as individual NSCs are not tracked over time. Complementarily, intravital imaging allows direct longitudinal tracking but generally does not read gene expression to infer molecular progression (*8*, *13*, *15*, *50*). It now remains crucial, but a challenge, to integrate these different modalities into a comprehensive understanding of NSC behavior at the individual cell and population levels. In particular, it is unresolved whether self-renewal originates from stochastic fate decisions within the main lineage (*16*), identifies a sublineage within the NSC population (*13*, *50*), or is an upstream state in an NSC hierarchy (*14*, *17*). Supporting the latter hypothesis, recent mathematical models in the adult mouse hippocampus (*17*) and zebrafish pallium (*14*) predicted a hierarchical organization of NSCs into subpopulations of different dynamics and fate, where a reservoir/dormant NSC population feeds into a more active operational/resting population responsible for neuronal production. In the zebrafish, quantitative predictions of population size, activation rates, and division modes could further be inferred from the clonal data (*14*). Specifically, the reservoir was postulated to account for 61% of all NSCs, to display an activation rate γ_r_ = 0.007 days (doubling time of 97 days), and to be engaged in asymmetric NSC/NSC divisions generating one reservoir and one operational NSC. The operational pool was predicted to account for the remaining 39% of NSCs and, with γ_o_ = 0.023 days (doubling time of 30 days), to stochastically choose between the NSC/NSC, NSC/NP, and NP/NP division modes with a bias toward neurogenesis. Notably, the lineage progression model that we propose here qualitatively and quantitatively fits these predictions when *deltaA* negativity versus expression is used to sign the reservoir versus operational pools, respectively: *deltaA^neg^* NSCs make 80% of the total and divide in an asymmetric *deltaA^neg^*/*deltaA^pos^* NSC/NSC fashion with an average activation rate γ_neg_ = 0.0056 days (doubling time of 124 days), while *deltaA^pos^* NSCs (20% of NSCs) display NSC/NSC, NSC/NP, or NP/NP division modes (fig. S3D) with an overall average activation rate γ_pos_ = 0.0246 days (doubling time of 28 days) and a final neurogenic output. These comparable cell behaviors and figures of similar orders of magnitude solidify both sets of data toward a hierarchical organization of NSC dynamics. Together, these results stress the invaluable contribution of our approach to directly overlay, for the first time, three modalities of NSC heterogeneities: mathematical predictions, gene expression changes, and lineage progression. Such inclusive approaches set the stage for a comprehensive multimodal understanding of NSC population dynamics in vivo.

## MATERIALS AND METHODS

### Experimental model and subject details

#### Fish husbandry and lines

All animal experiments were carried out in accordance to the official regulatory standards of the department of Paris (agreement numbers C75-15-22 and A91-4772 to L.B.-C., N.D., and L.M.) and conformed to French and European ethical and animal welfare directives (project authorization from the Ministère de l’Enseignement Supérieur, de la Recherche et de l’Innovation to L.B.-C.).

All procedures relating to zebrafish (*Danio rerio*) care and treatment are conformed to the directive 2010/63/EU of the European Parliament and the council of the European Union. Zebrafish were kept in 3.5-liter tanks at 28.5°C and pH 7.4 water and in salinity-controlled conditions. They were maintained on a 14-hour light/10-hour dark cycle and fed three times a day with rotifers until 14 days after fertilization and with standard commercial dry food (Gemma Micro from Skretting*) afterward. All transgenic lines Tg(*gfap:eGFP*) (*36*), Tg(*gfap:dtTOMATO*) (*41*), Tg(*mcm5:eGFP*)^gy2^ (*28*), Tg(*4.5deltaA,GFP*) (*29*), and Tg(*gfap:hZO1-mKate2*) (this paper) were maintained in the *Casper* double mutant background (*roy^−/−^; nacre^−/−^*) (*43*). Heterozygosity of each transgene was respected upon crosses except for the Tg(*gfap:hZO1-mKate2*) line that is visible in an adult only with two copies or more (we have multiple insertion lines). Ages of the fish are explicitly stated in the respective experiments. All fish were euthanized in ice-cold water (temperature below 4°C) for 10 min.

### Method details

#### Generation of the transgenic line Tg(gfap:hZO1-mKate2)

The *hZO1short-linker-mKate2* sequence was assembled using the NEBuilder HiFi DNA Assembly Cloning Kit (NEB), optimized for a three-fragment reaction following the manufacturer’s instructions. *hZO1short* (encoding human ZO1 protein lacking its actin-binding domain) followed by a linker and *mKate2* (*56*) fragments were cloned by polymerase chain reaction from plasmid *pYFP-N1_SP6_ZO1-short* (a gift from H.-G. Belting’s and M. Affolter’s laboratories) and *pmKate2-f-mem* (Evrogen), using the Phusion High-Fidelity DNA Polymerase (Thermo Fisher Scientific). To generate the transgenic line, the multisite Gateway technology (Thermo Fisher Scientific) was used, taking advantage of Tol2kit plasmids (*57*). *hZO1-mKate2* was first recloned into the *pME-MCS* plasmid (Tol2kit #237) by Eco RI and Bcu I digestions and then recombined with *p5E-GFAP* and *p3E-polyA* (Tol2kit # 302) in *pDestTol2CG2* backbone (Tol2kit #395, containing the transgenesis marker *cmlc2:egfp-polyA*). We performed microinjection of the constructs into one-cell *Casper* embryos together with transposase-capped mRNA (40 ng/μl). F0 adults crossed with *Casper* fish were screened for transmission of fluorescence (cardiac GFP and mKate2) in F1 embryos to generate a stable line. We chose to work with a multiple insertion line based on the quality and intensity of the signal obtained on adult fish with a 2-photon microscope.

#### Time lapses

Anesthesia was initiated by soaking the fish for 2 to 5 min in water containing 0.02% MS222 (Merck). They were then transferred into a water solution of 0.005% (v/v) MS222 and 0.005% (v/v) isoflurane to maintain the anesthesia during the whole duration of the imaging session (*28*). Overall, fish were anesthetized for about 30 min per session, and the recovery time (in freshwater without any drugs) after a session was less than 5 min.

#### Immunohistochemistry

Brains were dissected in 1× solution of phosphate-buffered saline (PBS) at a temperature of 4°C and directly transferred to a 4% paraformaldehyde solution in PBS for fixation. They were fixed for 2 to 4 hours at room temperature under permanent agitation. After four washing steps in PBS, brains were dehydrated through 5- to 10-min series of 25, 50, and 75% methanol diluted in 0.1% Tween-20 (Merck) PBS solution and kept in 100% methanol (Merck) at −20°C. Rehydration was performed using the same solutions, and then brains were processed for whole-mount IHC. After rehydration, the telencephali were dissected out and subjected to an antigen retrieval step using Histo-VT One (Nacalai Tesque) for 1 hour at 65°C. Brains were rinsed three times for at least 10 min in a 0.1% dimethyl sulfoxide (DMSO) and 0.1% Triton X-100 (Merck) PBS 1× solution (PBT) and then blocked with 4% normal goat serum in PBT (blocking buffer) for 4 hours at room temperature on an agitator. The blocking buffer was later replaced by the primary antibody’s solution (diluted in blocking buffer), and the brains were kept overnight at 4°C on a rocking platform. The next day, brains were rinsed 5 to 10 times over 24 hours at room temperature with PBT and incubated in a solution of secondary antibodies diluted in PBT overnight, in the dark, and at 4°C on a rocking platform. After three rinses in PBT over 4 hours, brains were transferred into PBS. Dissected telencephali were mounted in PBS on slides using a 0.7-mm-thick holders. The slides were sealed using Valap, which is a mixture of Vaseline (Merck), paraffin (Merck), and lanolin (Merck).

Primary antibodies were used at a final concentration of 1:500 for glial fibrillary acidic protein (GFAP) and PCNA, 1:250 for DsRed, and 1:200 for Sox2 and ZO1. Secondary antibodies were all used at a final concentration of 1:1000.

#### Image acquisition

Images of whole-mount immunostained telencephali were acquired on a confocal microscope (LSM700 Zeiss) using a 40× oil objective. We acquired images with a z-step of 0.65 μm. We averaged each line four times with an image resolution of 1024 × 1024 pixels with a bit depth of 12 bits. The power of the lasers was kept constants for all the acquisitions, and the GAIN (the voltage of the photomultipliers) was adjusted for each experiment. We recorded mosaics with a 15% overlap to image an entire hemisphere per fish.

Intravital imaging was performed on a dual-beam two-photon microscope (TriM Scope II, LaVision BioTec) with a 25×, 1.05–numerical aperture water immersion objective (Olympus). The mKate2 fluorophore [Tg(*gfap:hZO1-mKate2*)] was sequentially excited at 1120 nm with an optical parametric oscillator (80 MHz, ~100- to 200-fs pulses after the objective, Insight DS+ from Spectra-Physics), and the GFP fluorophore [Tg(*deltaA:eGFP*)] was excited at 950 nm with a titanium:sapphire oscillator (80 MHz, ~100 to 150 fs, Mai Tai HP from Spectra-Physics). The mean powers after the objectives were about 30 to 40 mW at 1120 nm and 9 mW at 950 nm. Emitted photons were split with a dichroic mirror (Di02-R561-25x36, Semrock) and detected with two GaAsP detectors (H7422-40, Hamamatsu). Each line was averaged two times and acquired sequentially for each laser to avoid cross-talk at the emission (pixel dwell time of 2.42 μs). Images were acquired with a field of view of 520 × 520 μm and spanning a depth range of 150 to 200 μm (voxel size of 0.29 μm by 0.29 μm by 2 μm). The initial plane of imaging was located approximately 150 μm underneath the skin surface.

#### Pharmacological treatment

Inhibition of Notch signaling was performed using the LY γ-secretase inhibitor (Merck). The stock solution of LY at 100 μM in DMSO was prepared and stored at −20°C.

For treatment, LY was applied in the fish swimming water at a final concentration of 10 μM (*7*). The solution was renewed every 24 hours. Control fish were treated with the same final concentration (0.1%) of DMSO carrier.

#### Overexpression of Shroom3

To generate the constructs used in overexpression experiments, the multisite Gateway technology was used, taking advantage of Tol2kit plasmids (*57*). To build the Shroom3-mCherry plasmid, *p5E-CMV/SP6* (Tol2kit#382), *pME-Shroom3*, and *p3E-mCherrypA* (Tol2kit#388) plasmids were recombined in pDestTol2pA2 (Tol2kit#394). For the negative control, *p5E-CMV/SP6* (Tol2kit#382), *pME-mCherry* (Tol2kit#386), and *p3E-pA* (Tol2kit#302) plasmids were recombined in pDestTol2pA2 (Tol2kit#394).

#### Electroporation of deltaA-MO

To selectively knock down *deltaA*, we electroporated fluorescein-tagged translational *deltaA* MO (GeneTools, Philomath, OR, USA) into NSCs of the adult pallium in vivo. The chosen *deltaA*-MO sequence was 5′-CTTCTCTTTTCGCCGACTGATTCAT-3′ as in (*44*). We compared the effect after 3 days with cells electroporated with a control generic fluorescein-tagged MO: 5′-CCTCTTACCTCAGTTACAATTTATA-3′. Both MOs were co-electroporated at 450 μM together with 225 ng/μl of the plasmid Tol2kit #228.

#### Ventricular microinjections and electroporations

Microinjections into the adult pallial ventricle were performed on anesthetized fish as described (*58*). For plasmid electroporations, plasmid DNA was diluted to a final concentration of 500 ng/μl in 1× PBS and injected into the ventricle. Fish were then administered four electric pulses (50 V, 50-ms width, 1000-ms space). Fish were sacrificed 3, 5, or 14 days after the injections.

#### Processing and analysis of IHC images

All of our analyses were performed on images with three channels: one channel with the AA staining (ZO1), one channel with nuclear staining (PCNA), and one channel with cytoplasmic staining (either *deltaA* or gfap). Using Fiji, we performed for all channels a three-dimensional (3D) median filter, a max Z projection, and a subtract background (rolling ball radius of 50 pixels). To automatically segment AA staining with a low rate of error, we performed a flat-field correction on this channel only to efficiently set a homogeneous intensity (our channel of interest was divided by the duplicated version of the same channel modified with a sizeable Gaussian blur of >30). In the same purpose and if needed, we also performed an enhanced local contrast filter (CLAHE, blocksize = 20). Cell contours were determined, and individual cells were identified using a seed-based region growing algorithm, followed by several rounds of manual corrections. Nuclear and cytoplasmic stainings were detected in single cells using the 20 to 30% highest pixel values (pixel values were ordered from least to most significant, and the range of the 20% highest value was selected using a given percentile as a threshold) followed by manual corrections. All these analyses are done with a custom MATLAB script (Y. Belaïche).

#### Processing and analysis of time-lapse images

We mounted the movies composed of z-stacks with two channels (ZO1 and *deltaA*) taken on different days using a custom Fiji macro. We performed translational and rotational spatial registration manually using the correct drift function on Imaris software. Then, we used the CSBDeep toolbox to denoise the apical membrane channel (ZO1). CSBDeep is a content-aware image restoration (CARE) that requires to be trained with a set of high- and low-resolution images. CARE is an open-source Python algorithm (*59*), and it has been adapted and trained with images from the notum of the *Drosophila* embryo. The restored channel, together with the second channel (*deltaA*), is MAX projected in 2D.

To improve the tracking, we first applied slight deformations over the image frames using the Image J’s plug-in bUnwarpJ (*60*). The information of all image channels was used to compute the transformation matrix between two consecutive frames. We chose the middle frame as the reference and aligned all the other frames from the referenced frame. The transformation matrices were saved in text files and served as registering other movies and performing the revert transform. The segmentation and tracking of the cell contours and *deltaA* from the registered image frames were done in Tissue Analyzer (*61*). We then aligned the segmented apical surfaces back to the preregistration state to compute their original apical areas. The revert transform of the image frame was deduced from its transformation file. We provide the scripts that wrap up all the steps. Dividing and nondividing tracks are processed separately, and further analyses are performed on R, PRISM, and Python (tabular dataset are also provided).

### Quantification and statistical analysis

The same experimenter carried out all the segmentation and corrections for a given experiment. Balanced ratios of females and males were included in the different experimental groups as much as possible.

For the IHC and intravital dataset, means and medians are per hemisphere per animal. The size of the analyzed regions differs from fish to fish. Thus, we collected different numbers of tracks per fish (data available on Zenodo). Statistical analyses were carried out using PRISM, R, and Python. The normality of the residuals of the responses was assessed using normality probability plots, and the homogeneity of the variance was inspected on a predicted versus residual plot. Nonparametric tests were used when the responses show a deviation of the residuals from a normal distribution and/or heterogeneous variances. In that case, overall effects were assessed with a Kruskal-Wallis test and all pairwise comparisons with the Mann-Whitney-Wilcoxon test comparing if two independent samples were selected from populations having the same distribution. All statistical analyses performed were two-tailed, and their significance level was set at 5% (α = 0.05).

#### Logistic regressions

##### Data sources and modeling process

Each NSC track was labeled individually according to whether the NSC underwent division during the study period. For NSCs that divided, their AA and *deltaA* expression before the division tp were recorded, while for NSCs that did not divide, the average AA and most prevalent *deltaA* expression (off or on) over the study period were used. For NSCs that divided, the *deltaA* expression of the two daughter cells was also tracked, with three possible values: *deltaA^neg^*/*deltaA^pos^* (for an asymmetric division), *deltaA^pos^*/*deltaA^pos^*, or *deltaA^neg^*/*deltaA^neg^*.

Estimation of the proliferative capacity and fate of the NSCs was carried out using logistic regression models, with AA and *deltaA* expression as covariates. To account for heterogeneity between fish and between treatments [wild type (WT) versus LY], the models were adjusted by adding fixed effects to the intercept and testing for interactions with other covariates. The identifiability of these fixed effects was ensured by the inclusion of two fish in both treatments. Inclusion and exclusion of parameters in the models were performed according to type II Wald χ^2^ tests.

##### Model for the proliferative capacities of NSCs

Individual tracks (1106; 844 from WT and 262 from LY) were used in this model, accounting for 214 divisions (125 from WT and 89 from LY). Both *deltaA* expression and AA, as well as an interaction term between these two covariates, were statistically significant in predicting NSC proliferative capacity. The model also had to be adjusted with an interaction term between treatments and *deltaA* expression.

As tracks were recorded over different time periods for the two treatments (around 35 days for WT and 4 days for LY), the probabilities of division estimated by the models correspond to an NSC tracked for the corresponding lengths of time. To directly compare the two estimates, we rescaled the estimate for WT to the shorter 4-day time period by assuming memory lessness of the NSC division process in WT fish: In this case, noting *p_k_* the probability of an NSC dividing over a time period of *k* days, we can relate the probability of not dividing for *k* days, (1 − *p_k_*), with that of not dividing for 1 day, (1 – *p*_1_),

$$1-p_{k}={\left(1-p_{1}\right)}^{k}$$

which yields a simple transformation to scale the probabilities to the same time frame,

$$p_{4}=1-\exp\left\{\frac{4}{35}\log(1-p_{35})\right\}$$

##### Model for the proliferative fate of NSCs

As NSCs expressing *deltaA* almost exclusively yielded *deltaA^pos^*/*deltaA^pos^* divisions (83 *deltaA^pos^*/*deltaA^pos^* versus 1 *deltaA^neg^*/*deltaA^pos^* in WT and all *deltaA^pos^*/*deltaA^pos^* in LY), only NSCs not expressing *deltaA* were used in the fit of the model. This accounted for 110 NSC divisions (41 from WT and 69 from LY). As there are three modalities for the *deltaA* expression of the daughter cells (*deltaA^neg^*/*deltaA^pos^*, *deltaA^pos^*/*deltaA^pos^*, and *deltaA^neg^*/*deltaA^neg^*), a multinomial log-linear regression model, rather than a logistic, was fitted. Type II Wald tests revealed that no parameter other than a fixed effect on the intercept accounting for the heterogeneity between fish was statistically significant in predicting the proliferative fate of the NSCs.
